# Methodology: *ssb*-*MASS*: a single seed-based sampling strategy for marker-assisted selection in rice

**DOI:** 10.1186/s13007-019-0464-2

**Published:** 2019-07-24

**Authors:** Juan David Arbelaez, Erwin Tandayu, Maria Ymber Reveche, Annalhea Jarana, Petra van Rogen, Line Sandager, Patrik Stolt, Enghwa Ng, Rajeev K. Varshney, Tobias Kretzschmar, Joshua Cobb

**Affiliations:** 1 International Rice Research Institute, Metro Manila, DAPO Box 7777, Los Baños, 1301 Philippines; 2 Intertek - AgriTech, 2-230-53 Alnarp, Elevenborgsvägen Sweden; 3 International Crops Research Institute for the Semi-Arid Tropics (ICRISAT), Patancheru, Telangana India; 4 Southern Cross Plant Science, Southern Cross University, PO Box 157, Lismore, NSW 2480 Australia

**Keywords:** Seed DNA extraction, Single nucleotide polymorphism (SNP), Rice (*Oryza sativa* L.), Marker-assisted selection (MAS), Forward breeding, Breeding, Genotyping, Rapid generation advancement

## Abstract

**Background:**

Integrated breeding approaches such as combining marker-assisted selection and rapid line fixation through single-seed-descent, can effectively increase the frequency of desirable alleles in a breeding program and increase the rate of genetic gain for quantitative traits by shortening the breeding cycle. However, with most genotyping being outsourced to 3rd party service providers’ nowadays, sampling has become the bottleneck for many breeding programs. While seed-chipping as prevailed as an automatable seed sampling protocol in many species, the symmetry of rice seeds makes this solution as laborious and costly as sampling leaf tissue. The aim of this study is to develop, validate and deploy a single seed sampling strategy for marker-assisted selection of fixed lines in rice that is more efficient, cost-effective and convenient compared to leaf-based sampling protocols without compromising the accuracy of the marker-assisted selection results.

**Results:**

Evaluations replicated across accessions and markers showed that a single rice seed is sufficient to generate enough DNA (7–8 ng/μL) to run at least ten PCR trait-markers suitable for marker-assisted selection strategies in rice. The DNA quantity and quality extracted from single seeds from fixed lines (F_6_) with different physical and/or chemical properties were not significantly different. Nor were there significant differences between single seeds collected 15 days after panicle initiation compared to those harvested at maturity. A large-scale comparison between single seed and leaf-based methodologies showed not only high levels of genotypic concordance between both protocols (~ 99%) but also higher SNP call rates in single seed (99.24% vs. 97.5% in leaf). A cost–benefit analysis showed that this single seed sampling strategy decreased the cost of sampling fourfold. An advantage of this approach is that desirable genotypes can be selected before investing in planting activities reducing the cost associated with field operations.

**Conclusion:**

This study reports the development of a cost-effective and simple single seed genotyping strategy that facilitates the adoption and deployment of marker-assisted selection strategies in rice. This will allow breeders to increase the frequency of favorable alleles and combine rapid generation advancement techniques much more cost-effectively accelerating the process and efficiency of parental selection and varietal development.

**Electronic supplementary material:**

The online version of this article (10.1186/s13007-019-0464-2) contains supplementary material, which is available to authorized users.

## Background

Plant breeding programs producing inbred lines have two concurrent goals: (i) identifying parents for subsequent breeding cycles, and (ii) identifying new inbreds for varietal release [18]. Reducing the time to complete both activities is an effective way to increase the rate of genetic gain and efficiently deliver new varieties to farmer’s fields [30]. Integrated breeding approaches such as the combination of marker-assisted selection (MAS) and rapid line fixation (either through single seed decent (SSD; [6, 28] or double haploid creation (DH; [22] can be used to both increase selection efficiency and shorten the breeding cycle [35, 36]. In marker-assisted selection (MAS), molecular markers associated with favorable large-effect alleles are used as indirect selection criteria to improve breeding populations by deterministically increasing the frequency of specific high-value haplotypes in the breeding program [2, 29]. Rapid generation advance using SSD is an easy and cost-effective way to quickly attain line homozygosity in rice and effectively reduce the duration of variety development. In particular for self-pollinated crops, SSD methods are often cheaper than doubled haploid technologies since the later not only required high level of technical expertise but sophisticate tissue culture laboratories and facilities to generate large numbers of double haploid lines. The use of simple agronomic interventions can encourage early flowering [14, 35] reducing even further the time required in SSD methodologies to generate fixed lines. It’s common in many public plant breeding programs to impose marker assisted selection in the F_2_ generation in order to reduce the number of selection candidates handled by the program, however, closer to fixation (e.g. F_6_ or S_6_) the frequency of desired homozygous genotypes for two unlinked target loci more than triples (increasing from 0.0625 in the F_2_ to 0.235 in the F_6_ population). Thus, to identify 50 lines with the desired homozygous genotypes at two loci, on average 800 F_2_ individuals would be required compared to only 213 in the F_6_ population. As a consequence, the cost of genotyping is substantially reduced when MAS is conducted closer to line fixation. This is especially relevant as adding additional MAS targets increases population sizes exponentially. Further, leaf sampling F_2_ individuals is required as each plant is genetically unique and F_2_ derived seeds maternal and embryonic tissue DNA profiles are still segregating. However, imposing MAS at the F_6_ generation permits the use of whole seed sampling for genotyping with minimal risk of failure because each seed on the resulting panicle is nearly genetically identical.

In the International Rice Research Institute’s (IRRI) irrigated varietal rice breeding program a typical integrated rapid-line-fixation and MAS-based forward-breeding strategy begins by developing a breeding population from parents polymorphic for alleles that are targeted for MAS. This cross is quickly converted to an F_6_ recombinant inbred line population within 2 years via SSD [14]. Each F_6_ line is grown in the field as row, barcoded, and 60 days after transplanting leaf tissue is collected and shipped to a 3rd party genotyping service provider for evaluation. Uniform leaf-discs are excised from each leaf sample, dried, and shipped to Intertek-Agritech (http://www.intertek.com/agriculture/agritech/) where DNA is isolated and marker assays targeting a number of disease resistance and grain quality traits (http://gsl.irri.org/) are surveyed. Allele specific fluorescence scores are converted to allele calls and the data is sent to IRRI for interpretation and decision-making. Selected plants are then harvested and processed for subsequent yield trials. While the use of uniformly sized leaf discs helps ensure genotype data quality, when sampling thousands of lines in a short period of time, field collection, processing, and tracking of leaf tissue samples is considered one of the most limiting factors in deployment of large-scale MAS strategies in many breeding programs [17].

Seed-based sampling strategies have proven to dramatically increase efficiency and reduce cost in commercial maize breeding programs by combining seed-chipping technologies with MAS on doubled haploid (DH) lines for simply inherited genes with large-effect alleles [32]. Seed-based genotyping efforts have been tested in public breeding programs for maize [17], rice [10, 24], barley [34], soybean [4], and cotton [37]. Seed-based genotyping enables selections to be carried out in advance of more expensive planting or off-season activities. It also makes tissue sampling more automatable, saving field and operational costs, which can be re-allocated for other breeding activities. Through automated barcode systems, seed samples can more easily be manipulated and tracked between tissue source and genotype data, further minimizing error. In addition, seed samples can be physically handled more easily and stored at room temperature with less risk of spoilage.

As more rice breeding programs adopt single seed descent methodologies (from F_2_ to F_6_) in their breeding strategies [3, 12, 14], novel seed-based sampling protocols can greatly increase the efficiency and reduce the cost associated with MAS activities. Concerns related with DNA quality, quantity, and sources of genotyping errors that might be caused by seed-based genotyping in a large-scale rice-breeding program, to our knowledge, have not been investigated. Therefore, the main objectives of this study are to: (i) Examine if DNA quantity and quality extracted from a single seed with different physical and/or chemical properties and sampled at different developmental stages of maturity generates sufficiently accurate results for MAS; (ii) Determined the rate of genotypic concordance between single-seed-based and leaf-based sampling strategies; (iii) Establish and validate a large-scale single seed-based MAS strategy (ssb-MASS) for forward -breeding in a varietal development breeding program; and (iv) estimate the efficiency and potential economic advantages of ssb-MASS compared to a conventional leaf-based sampling protocol. We report the development, validation, and scaling of a single seed-based MAS strategy for forward-breeding that increases the efficiency of MAS in varietal rice breeding programs, reduces the cost associated with MAS activities, and does not compromise the quality of the genotypic data.

## Results

### Single seed-based genotyping of rice grains with different physical/chemical properties

**Fig. 1:**
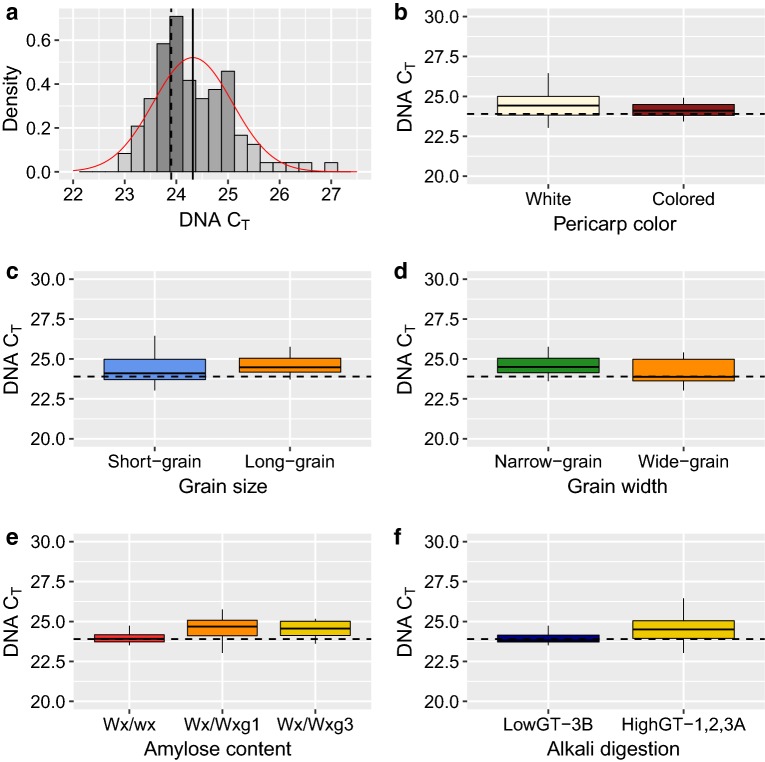
C_T_ values distribution and comparisons between single seed samples with varying physical and chemical properties. **a** Distribution of C_T_ values for DNA from 96 different single seed samples in rice. The average C_T_ value on rice seeds (C_T_ = 24.3) is illustrated with a solid line and the average C_T_ value obtained using leaf tissue (C_T_ = 23.9) is illustrated by a dashed line. Analysis of variance among seeds with different **b** pericarp color, **c** grain size, **d** grain width, **e** active amylose-content, and **f** alkali digestibility. The average C_T_ value estimated in leaf tissue (C_T_ = 23.9) is illustrated in each boxplot by a dashed line and the acronym ‘n.s’ indicates a non-significant difference between classifications

To test if a single F_6_ seed yielded sufficient DNA to run at least ten independent SNP KASP assays, C_T_ values from 96 seed samples from 24 rice accessions were generated and analyzed. The average C_T_ value observed among the single seed samples was 24.3 (with standard deviation (SD) of 0.76) and ranged from 23 to 27 (Fig. 1a). The average C_T_ value obtained from 12 independent leaf tissue samples was 23.9, which is equivalent to 7–8 ng/μL (Fig. 1a, Additional file 1: Table S3). No significant differences were observed between the C_T_ values from DNA extracted from leaf and mature seed samples (Fig. 1a, Additional file 1: Table S3).

Comparison on C_T_ values between accessions with different physical and chemical grain properties was done to evaluate the potential impact these properties have on DNA concentration as judged by C_T_ values (Fig. 1b–f, Additional file 1: Table S4). Grains with different pericarp color, size, width, amylose content and alkali digestibility were compared. An analysis of variance showed no significant differences between C_T_ values obtained from grains with different physical or chemical properties (Fig. 1b–f, Additional file 1: Table S4).

**Fig. 2:**
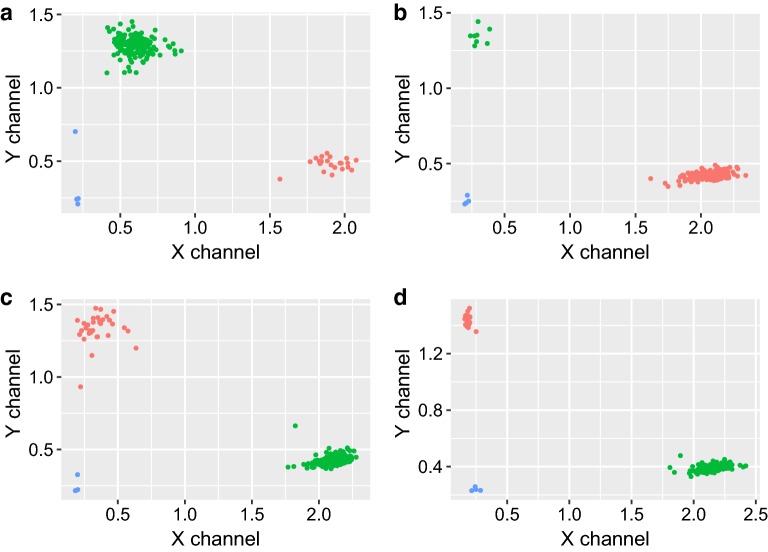
Kompetitive allele specific PCR cartesian plots. HEX™ (Y channel) versus FAM™ (X channel) intensity scatter plots for the KASP assay **a** snpOS002, **b** snpOS0006, **c** snpOS0007b, and **d** snpOS0015 associated with the loci *xa13*, *Pi*-*ta*, *Pi9*, and *Pi54* respectively. Each panel contains results of the SNP assay for 96 single seed samples. Blue dots represent negative controls; green and red dots represent different alleles of each marker

Genotyping results aggregated across nine KASP assays showed an average SNP call rate of 98.38%, higher than the minimum 95% SNP call rate required to consider a genotyping project successful. Cartesian bi-plots for each KASP assay were created by plotting the fluorochrome dye intensity values which are commonly used to determine the allelic discriminatory capacity of each SNP assay (Fig. 2a–d). High quality allelic discrimination was observed among all SNP markers utilized in this study (Fig. 2a–d).

### Evaluation of single seed-based sampling at different developmental stages

To estimate the potential impacts of seed developmental stage on genotype data quality, F_6_ seeds harvested at 7, 15, 25, and 30 DAPI were subjected to DNA extraction and genotyped alongside standard leaf tissue sampling using ten KASP assays (Additional file 1: Table S5).

**Fig. 3:**
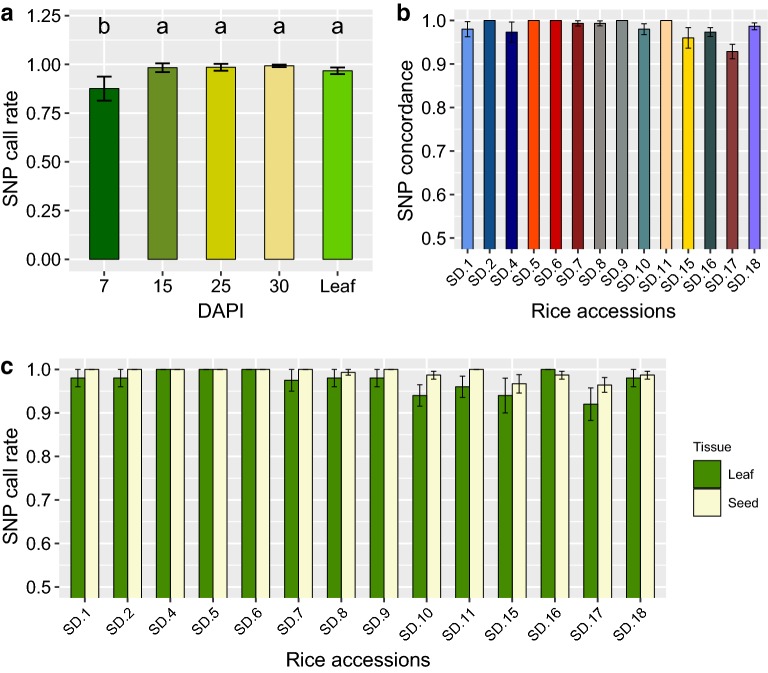
SNP call rates and accuracies. **a** SNP call rates on single seed samples harvested 7, 15, 25, and 30 days after panicle initiation (DAPI), and leaf tissue. Each bar represents fourteen accessions replicated 5 times for each of ten independent KASP assays. **b** Genotypic concordance among single seed samples collected at 15, 25, and 30 DAPI relative to a reference sample generated from the leaf tissue. Each bar represents concordance rates for all 14 different accessions. **c** Call-rates for each SNP measured on 14 different accessions including leaf and single seeds samples collected at 15, 25, and 30 DAPI

The average call rates for the single seed sampling strategy across all accessions harvested at 7, 15, 25, and 30 DAPI were 87.6%, 98.3%, 98.5%, and 99.3% respectively (Fig. 3a). The average call rate for leaf tissue was estimated to be 97% (Fig. 3a). A Tukey-HSD multiple comparisons test showed no significant differences between genotypic call rates generated at 15, 25, 30 DAPI, and leaf tissue. Call rates for samples collected at 7 DAPI were significantly lower than the other developmental stages (Fig. 3a).

Single seed samples collected at 7 DAPI were removed from the analysis, and call rates were estimated for each of the 14 rice accessions using the remaining genotypic data. The average SNP call rate among the seed samples was 99.18%, ranging from 96.5 to 100%. These values were slightly higher than those observed for leaf tissue samples which averaged 97.4% and ranged ranging from 92 to 100%. This same pattern was observed when average call rates for seed and leaf samples disaggregated by accession were compared side by side (Fig. 3c). The average percentage of genotypic concordance between single seed and leaf tissue samples for each accession was 98.35% and ranged between 92.86 and 100% (Fig. 3b). Rice accessions SD.17 and SD.15 showed the lowest concordance rates (93% and 96% respectively) among the 14 lines and also showed the lowest SNP call rates (96.4% and 96.7%) (Fig. 3b, c).

### Single seed-based MAS results vs. conventional leaf sampling at scale

**Fig. 4:**
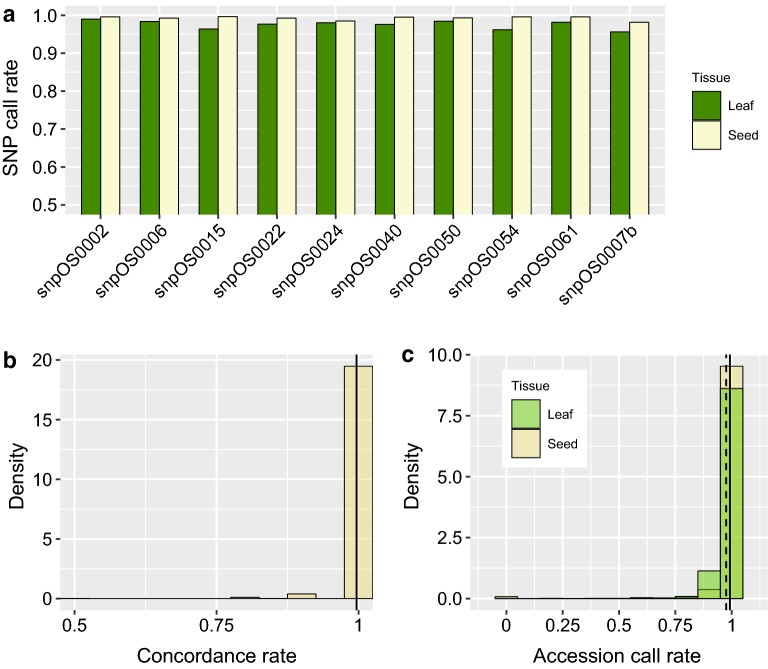
Single seed-based sampling for MAS at scale. **a** Call rates from each SNP assay estimated using 1466 single seed and leaf samples. **b** Distributions of genotypic concordance for all 1466 accessions between single seed and leaf-based sampling strategies. **c** Call rate distribution across single seed and leaf samples for 1466 rice lines. Solid and dashed lines show the average call rates of 99.24% and 97.5% estimated on single seed and leaf-based samples respectively

Large-scale single seed and leaf-based sampling strategies were compared using 1466 fixed lines (F_6_ plants) from IRRI’s irrigated breeding program. Single seed and leaf-based sampling strategies were conducted simultaneously on the same 1466 plants and genotyped with the same ten KASP assays (Additional file 1: Table S6). The average call rate estimated across the ten SNPs from the single seed-based method was 99.24% ranging from 98.16 to 99.66% (Fig. 4a). The call rates were not significantly different from those obtained from the leaf-based protocol. With an average of 97.54% (ranging between 95.63 and 98.98%) the conventional leaf protocol showed slightly lower call rates compared to the single seed-based results (Fig. 4a). The average percentage of genotypic concordance among the 1466 samples was 99% when individual markers were compared (Fig. 4b). The concordance rate between both protocols estimated by marker calls generated from all ten SNPs was 98%. Out of the 30 samples that had at least one miss matched genotype call, 21 of them were due to miss-called heterozygous genotypes, ten observed on single seed samples and eleven on leaf samples, respectively. When call rates across the ten SNPs were estimated individually for each accession an average call rate of 99.24% was observed in the single seed strategy versus 97.5% for the leaf-based MAS methodology (Fig. 4c).

**Fig. 5:**
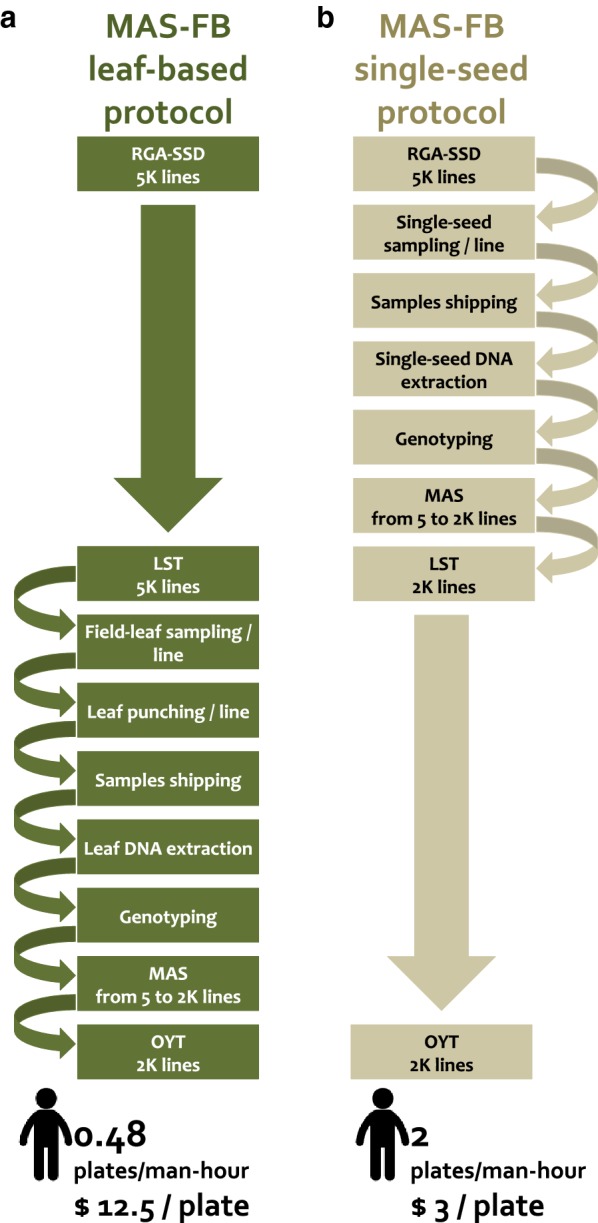
Cost comparison between seed and leaf sampling based MAS forward breeding strategies on fixed lines. **a** Conventional leaf-based sampling protocol, versus **b** single seed-based sampling schemes

An analysis of cost effectiveness between the single seed-based and conventional leaf-based protocols was carried out for the 1466 field collected samples (Fig. 5, Additional file 2). In the conventional leaf-based protocol, 15.5 and 17.5 man-hours were necessary to harvest and leaf-punch 16 plates with 1466 leaf samples (94 samples/plate), respectively. In total, the conventional leaf-based sampling protocol required 33 man-hours to fully process 16 plates with 1466 samples. On average this represents 0.48 plates processed per man-hour. In the modified single seed-based strategy only 8 man-hours were necessary for processing the same number of samples. The total amount of man-hours in the modified single seed-based strategy is implemented in collecting and placing individual seeds into their corresponding plate well. On average this represents 2 plates processed per man-hour, a fourfold increase in efficiency per man-hour compared with the leaf-based protocol. With an estimated fixed cost wage of $6 per hour, the cost of processing 1 plate with the conventional protocol was $12.5 while the same number of samples was processed for $3 using the single seed-based strategy (Fig. 5, Additional file 2).

## Discussion

Molecular-markers are a useful tool for monitoring the presence of key qualitative and quantitative traits by quickly and cheaply surveying a few large effect loci [7]. Despite the success in identifying QTL controlling a wide variety of traits in different species and the identification of the functional variants underlying many of these QTLs [11], the success of MAS for major genes in public breeding programs has been limited [13, 27]. Economical, logistical, and technical considerations are among the factors that could constrain the full deployment of MAS strategies in many breeding programs. Increasing this efficiency and reducing the cost associated with MAS is key for the success of integrating different molecular strategies into plant breeding programs.

To be effective a high-throughput MAS system requires simple and rapid DNA extraction methods [34] to be combined with simple and effective sampling strategies that permit high quality data return and tracking of information. In this study a high-throughput single seed-based sampling method was compared to a conventional leaf-based sampling method using DNA extraction protocols developed at Intertek-AgriTech (http://www.intertek.com/agriculture/agritech/) for routine MAS applications in rice. The reported single seed-based sampling strategy for forward breeding applications of MAS should also be compatible with different DNA extraction protocols developed for rice in different studies [16, 24, 26, 31]. The quality and quantity of DNA obtained from these methods though should be validated as outlined in this study in order to ensure suitability for MAS deployment.

Based on observed C_T_ values and genotypic results, the average amount of DNA extracted from a single seed (~ 7.5 ng/μL) was sufficient for genotyping at least ten KASP markers which is more than the typical number of loci targeted for MAS-based forward-breeding strategies in rice. The low standard deviation values observed for C_T_ values (SD = 0.76) demonstrated that the DNA concentration extracted from single seeds is uniform across samples and was not significantly affected by different grain physical or chemical properties, making it a suitable system for genetic evaluation of both elite and exotic germplasm. Additionally this study tested the effect of different seed developmental stages on genotypic data quality and found no effects on the quality of genotypic results from seeds harvested as early as 15 DAPI to physiological maturity as evidenced by average call rates of ~ 99%. This allows alternative tissue sampling protocols to be adapted for plants grown under field or greenhouse conditions. Likewise, a concordance rate of ~ 98% between the two protocols should bring confidence to breeding programs seeking to transition from leaf-based sampling to a single seed-based genotyping system.

Similarly, high call rates observed across the ten SNP KASP assays (~ 99%) and the high genotypic concordance results between single seed and leaf-based MAS strategies (~ 99%) in the large-scale project validated the feasibility of adopting this new protocol without diminishing the quality of genotypic results. The low rate of missing, uncallable, or erroneous data points observed in both protocols (< 1%) is valuable information that can be used to re-design MAS strategies in inbred rice breeding programs. Additionally, the cost-effectiveness analysis demonstrated that the implementation of a single seed-based MAS strategy for forward-breeding increases the efficiency of MAS activities by fourfold, and reduces labor costs four times when compared to the conventional leaf-based protocol. The efficiency of the single seed-based MAS strategy is increased since less time and labor is required to collect and placed singles seeds into their corresponding plate well compared to the time and labor required to harvest leaf-tissue follow by leaf punching activities to place the standardized leaf discs necessary in the conventional leaf-based protocol. The single seed-based methodology also decreases planting space and field costs by enabling MAS-based selections to take place before planting, and accelerates the data turn around time by eliminating the need to produce vegetative tissue prior to selection.

When the cost of genotyping is higher than those associated with generating a fixed line, MAS activities to determine homozygous genotypes for high priority traits in fixed lines are more cost-effective than in segregating generations. In this study we demonstrated that a single whole seed in rice can be effectively used for MAS, avoiding the complexity of seed chipping an asymmetrical rice seeds, and ensuring a high probability that the genotypic profile of the sampled seed matches the remnant seed from the same plant, as demonstrated by the high genotypic concordance rates reported in this study.

This strategy also has the potential to be extended beyond MAS applications and be implemented in different breeding operational activities such as pedigree verification and seed purity determination for seed inventory management and quality control methodologies in rice breeding programs. Continuous improvement on best seed management practices as well as crop breeding operational practices that rely on digitalization and implementation of appropriate database management system would minimize any potential risk associated with the integration of these strategies into different crop improvement programs.

The successful development of a cost-effective and simple single seed-based sampling strategy for rice has the potential to facilitate the adoption and utilization of MAS in public rice breeding programs, thus rapidly and cost-effectively increasing the frequency of high-value alleles when used in combination with a single seed descent line fixation strategy to accelerate the process of parental selection and varietal development.

## Methods

### Plant material and growing conditions

For evaluating the potential effects on the quantity and quality of DNA extracted from a single seed at maturity from genotypes with grains exhibiting different physical and chemical properties, 96 single seed samples from 24 different rice accessions replicated 4 times were sampled for DNA extraction and genotyped (Additional file 1: Table S1). These accessions were chosen based on well known remarks from previous studies, commonly used breeding checks in the IRRI irrigated breeding program and grain type classifications that differ by grain size, grain width, pericarp color, alkali digestibility and amylose content (Additional file 1: Table S1). Additionally, among the analyzed accessions donor lines for desirable alleles at well known grain quality genes were included to verify the genotypic data quality of ten commonly used single nucleotide polymorphism (SNP) Kompetitive Allele Specific PCR (KASP) assays used routinely in rice breeding.

The effect of seed developmental stage on the DNA quantity and quality was also compared between whole seed and leaf-based sampling strategies using a set of 14 accessions (Additional file 1: Table S1) replicated 5 times. For each accession a single panicle per plant was tagged at the day of its exertion and seeds were sampled at 7, 15, 25, and 30 days after panicle initiation (DAPI). These time points represent well described developmental phases in rice, namely: milky, dough, yellow-ripe, and maturity stages. At each time point, five seeds from one panicle per accession were sampled to make up 70 samples on a single deep-well plate. In addition, 5 leaf samples were also collected from each accession to be compared with the whole seed-based sampling results. Plants were grown in pots at IRRI’s Zeigler Experimental Station (ZES) screen house facilities in Los Baños, Philippines.

Additionally, an in situ large-scale comparison between the seed-based sampling strategy and the current routine leaf-based sampling protocol was conducted by sampling single seeds and leaf punches from 1466 F_6_ plants from the head rows in the line stage testing (LST) breeding class of IRRI’s irrigated lowland rice breeding program that are routinely prioritized for MAS (Additional file 1: Table S1). Lines in the LST class were grown in 2-row plots with 6 plants per row spaced 40 cm from each other at IRRI’s ZES field facilities in Los Baños, Philippines. During the implementation of each sampling activity, the number of workers, time spent on each step, and operational costs were recorded to compare the relative difference in cost-effectiveness between the two protocols.

### Tissue sampling and preparation

#### Single seed-based sampling strategy for MAS

For this protocol a single seed from each sample was collected from a barcoded seed envelope containing F_6_ seeds derived from a single F_5_ plant. Each seed envelope was scanned using a handheld Zebra Scanner (model DS3678, http://www.zebra.com) linked to the mobile application “Coordinate” (http://www.phenoapps.org) which simultaneously creates a 96-well plate layout and stores the line information in real time during sampling. A single seed was then collected from the envelope and placed in a 96-deep-well plate on the coordinates defined by the ‘Coordinate’ application. These steps are repeated for each sample until the whole plate is completed leaving the last two wells empty for negative/positive controls. The full plate was covered with a silicon cap mat or sticky paper and was shipped immediately to Intertek-AgriTech (http://www.intertek.com/agriculture/agritech/) for DNA extraction, marker assay and scoring. A detailed version of this protocol can be found in Additional file 2.

A modified version of the single seed-based sampling protocol was also developed for field conditions. In this version a single seed from one plant per line was collected by hand and placed in a 96-deep-well plate defined by the ‘Coordinate’ app. These steps were repeated for each line until the whole plate was filled leaving the last two wells empty for negative/positive controls. The full plate was then covered with a silicon cap mat or sticky paper and shipped immediately to Intertek-AgriTech for DNA extraction, marker assay and scoring.

#### Leaf-based sampling strategy for MAS

For the leaf-based protocol, leaf tissue was sampled from F_6_ rice lines derived from a single panicle of an F_5_ plant. The resulting F_6_ plants from each line were grown under field conditions and sampled 60 days after transplanting. Twelve F_6_ plants from each line were grown in the field and identified using a barcoded identification label. Each line’s barcode was scanned and synchronized with the “Coordinate” application. A waxed-paper envelope with the same barcode was also scanned and matched with the field barcode. Leaf tissue was collected from the first plant of each line and stored in waxed-paper envelopes. This process was repeated until all lines were sampled. The envelopes were taken to the lab and dried for 2 days at 50 °C using a convection oven or a lyophilizer. Envelopes containing dried leaf tissue were grouped in 94-sample batches to have uniform leaf-punches excised from the sample. To do this, dried tissue stored in the waxed-paper envelopes was taken out and four 4-mm diameter discs from each leaf sample were punched directly into a 96-deep-well plate using the AK-EP100 bench-top leaf puncher (Applied King, http://www.appliedking.com/lab-automation/) in the pre-defined order determined by the ‘Coordinate’ app. This last step was repeated for all samples until the batch was completed, leaving the last two wells empty as negative/positive controls. The plate was then covered with a silicon cap mat or sticky paper and shipped immediately to Intertek-AgriTech for DNA extraction, marker assay and scoring.

#### DNA isolation, quantification and genotyping

MAS activities at IRRI implement a DNA extraction protocol routinely used at Intertek-AgriTech (http://www.intertek.com/agriculture/agritech/) based on LGC oKtopure™ automated high-throughput ‘sbeadex™’ DNA extraction and purification system (https://www.biosearchtech.com/). The ‘sbeadex™’ system uses magnetic separation for the preparation of nucleic acids. The first step in this protocol involves homogenizing leaf or seed tissue samples by steel bead grinding in 96 deep-well plates. The grinded tissue is incubated with a DNA extraction buffer available in the plant DNA preparation ‘sbeadex™’ kit (https://www.biosearchtech.com/) from LGC. Finally, extracted DNA is purified using superparamagnetic particles coated with ‘sbeadex™’ surface chemistry that captures nucleic acids from a sample. Purified DNA is eluted and ready for use in downstream processes (https://www.biosearchtech.com/). The quantity and quality of extracted DNA was determined by running PCR on an endogenous rice gene via a real time PCR assay where a positive reaction is detected by the accumulation of a fluorescent signal that determines the cycle threshold or C_T_ value. The C_T_ value is defined as the number of cycles required for the fluorescent signal to cross a pre-determined threshold. As C_T_ values are inversely proportional to the amount of nucleic acid in the sample they serve as an accurate and quantitative assessment of DNA quantity that is appropriate for PCR. In rice with a genome size of 0.5 pg/genome, a C_T_ value of 24 approximately equals 15,000 genome copies per μL, which represents a DNA concentration of 7–8 ng/μL. All tissue samples used in this study were genotyped using a set of ten trait specific Kompetitive allele specific PCR (KASP, https://www.lgcgroup.com/) based SNP markers (http://gsl.irri.org/genotyping/trait-based-genotyping/10-snp-panel) developed by IRRI’s Genotyping Services Laboratory. These ten SNP markers are associated with key molecular breeding targets, including blast resistance genes *Pi9*, *Pita*, and *Pi54* [20], the bacterial blight resistance genes *xa5*, *xa13* and *Xa21* [15], the submergence tolerance gene *Sub1* [8, 33], the brown plant-hopper resistance gene *BPH17* [19], the grain chalkiness gene *Chalk5* [23] and the grain fragrance gene *BADH2* [21]. In the large-scale validation experiment the marker associated with *xa13* was replaced by the marker associated with resistance to rice tungro spherical virus 1 or *RTSV1* [1]; Additional file 1: Table S2).

### Statistical analysis

An analysis of variance (ANOVA) was used to test for significant differences in the C_T_ values of DNA extracted from leaf tissue and single seeds with contrasting physical or chemical grain properties. Custom R scripts (2018, R core development team) were used for calculating the call-rate and concordance between single seed and leaf-based genotype data. SNP call rates were calculated as the average proportion of successfully called genotypes for each SNP across all samples from different accessions and seed developmental stages. The SNP genotypic concordance rate was measured as the proportion of exact genotypic matches between identical SNPs genotyped on samples processed using the single seed and leaf-based tissue sampling protocols. ANOVA was calculated using the R function ‘*anova*’ [9]. Multiple comparisons were estimated using the Tukey’s HSD (honestly significant difference) [5] method using the R function ‘*HSD.test*’ from the R package ‘*agricolae*’ [25].

### Cost-effectiveness comparison between the single seed and leaf-based sampling protocols

To estimate the number of samples processed by one worker in 1 h, a total of 1466 samples (sixteen 96-deep-well plates) were collected and processed using the single seed and leaf-based sampling protocols. The total number of man-hours (*Total*_*Man–Hours*_) spent on each protocol was calculated by multiplying the number of workers that participated ($N_{workers}$) by the number of hours ($Hours$) spent on sampling and processing the tissue before DNA extraction (i.e. $Total_{Man - Hours} = N_{workers} \times Hours$). The number of plates per man-hour ($Plates_{Man - Hour}$), or the average number of plates that are processed in one man-hour, was estimated as the ratio of total man-hours invested in processing the samples for each protocol ($Total_{Man - Hours}$) against the total number of plates processed ($Plates_{Man - Hour} = Total_{Man - Hours} /16$). The cost associated for sampling one plate (containing 94 samples + 2 negative/positive controls) was estimated as the average hourly cost of labor based on estimated salary rates at IRRI for research labor.

## Additional files

**Additional file 1: Table S1.** List of accessions used in this study. **Table S2.** Information on the SNPs used in this study. **Table S3.** DNA C_T_ values for single seeds and leaf comparison. **Table S4.** Accessions, DNA C_T_ values and genotypic data generated in the physical-chemical analysis used in this study. **Table S5.** Accessions, and genotypic data generated in the seed developmental analysis used in this study. **Table S6.** Accessions, and genotypic data generated on 1466 rice lines for during a large-scale validation study.

**Additional file 2.** Single seed based sampling strategy protocol for rice.

## Data Availability

The datasets supporting the conclusions of this article are included within the article and in its additional tables and file.

## Abbreviations

SNP: single nucleotide polymorphism
MAS: marker assisted selection
IRRI: International Rice Research Institute
ZES: Zeigler Experimental Station
FB: forward-breeding
LST: line stage testing
C_T_: cycle threshold
KASP: Kompetitive allele specific PCR
ssb-MASS: single seed-based marker assisted selection strategy
