# Supplementary material for: Methodology: ssb-MASS: a single seed-based sampling strategy for marker-assisted selection in rice
Source: Plant Methods. 2019 Jul 24;15:78. doi: 10.1186/s13007-019-0464-2 (PMC6652012; doi:10.1186/s13007-019-0464-2)
Supplement: Supplementary file 2 — Additional file 2. Single seed based sampling strategy protocol for rice. [file 13007_2019_464_MOESM2_ESM.docx]

**SINGLE-SEED BASED MARKER ASSISTED SELECTION STRATEGY (*ssb-MASS*):**

**PROTOCOL FOR SAMPLING SEEDS STORED IN ENVELOPES**

Genotyping Services Laboratory

IRRI Service Laboratories

Integrative Research Support Platform

and

Rice Breeding Platform

March 2019


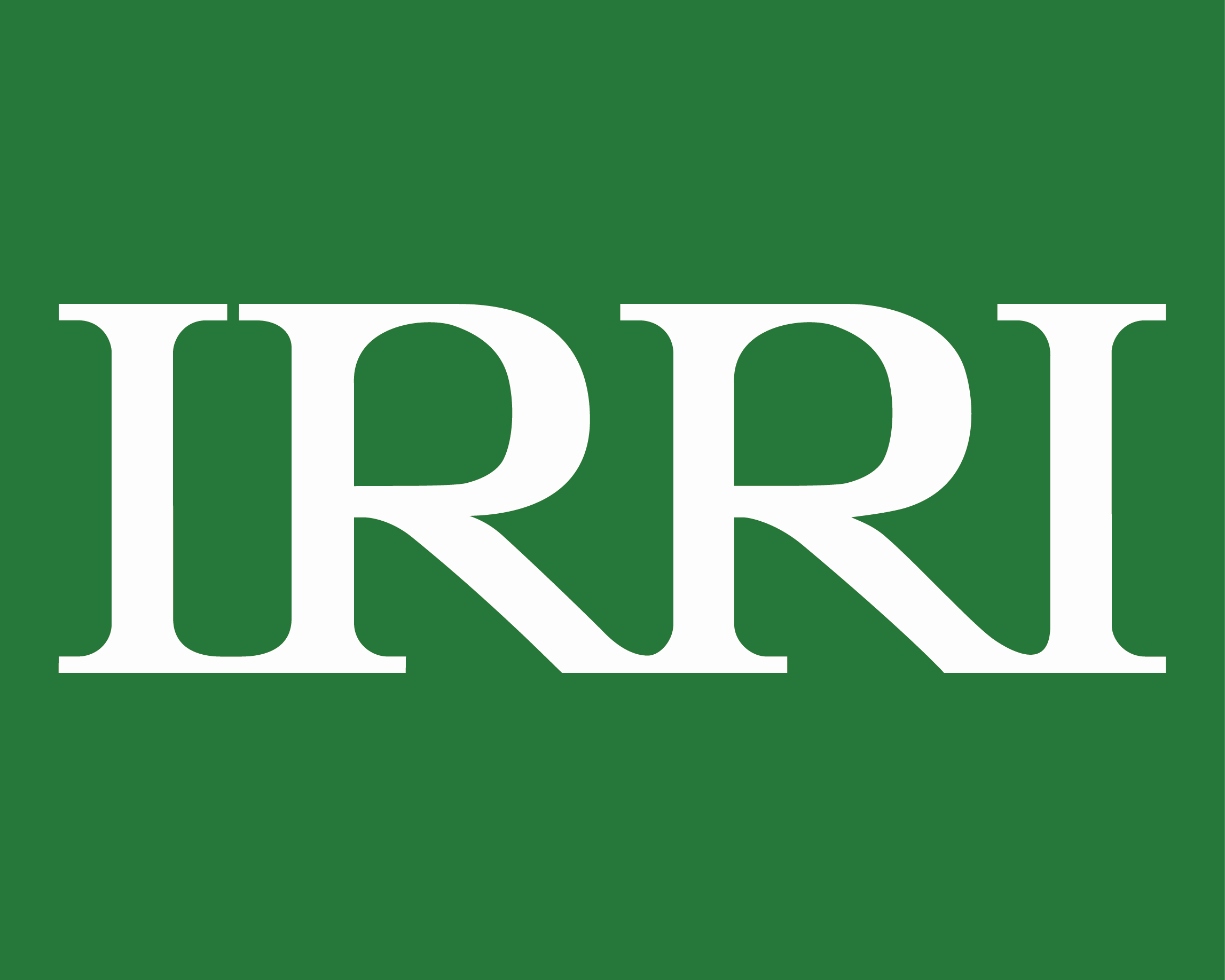


| **SINGLE-SEED BASED MARKER ASSISTED SELECTION STRATEGY (*ssb-MASS*):**  **PROTOCOL FOR SAMPLING SEEDS STORED IN ENVELOPES** |
| --- |

| **MATERIALS** | |
| --- | --- |
| 1 | Barcoded 96-deep-well plates specified by Intertek-Agritech or the genotyping service provider |
| 2 | Hand-held Zebra Scanner (Model: DS3678) or any barcode scanner that is compatible with your barcode labels |
| 3 | CT5 Rugged Phone or any android mobile phone or tablet compatible with ‘Coordinate’ app |
| 4 | 96-deep-well-plate cover mat |

| **PRE-SAMPLING REQUIREMENTS** | |
| --- | --- |
| 1 | Install ‘Coordinate’ ([www.phenoapps.org](http://www.phenoapps.org)) mobile app on the android mobile phone or tablet |
| 2 | Create sampling template using ‘Coordinate’ as specified in the app instructions ([www.phenoapps.org](http://www.phenoapps.org)) skipping wells on the H11 and H12 plate coordinates as determined by Intertek-Agritech (control wells). |
| 3 | Label and barcode seed envelopes and 96-deep-well plates. |
| 4 | Sort seed envelopes and 96-deep-well plates in the order intended for the seed sampling. |

| **PROCEDURES** | |
| --- | --- |
| 1 | Connect hand-held Zebra barcode reader and the mobile app via Bluetooth following manufacturer’s specification.  Note: Please see appendices A & B for Zebra Scanner Pairing barcodes |
| 2 | Open ‘Coordinate’ application on android device. |
| 3 | In the Coordinate application, create a new grid. Coordinate will prompt the user to input the plate name and the username. Alternatively, the user may scan these information from the appropriate barcodes (Plate Barcode & Username Barcode) Note: See Appendix A for step-by-step instructions |
| 4 | Scan envelope barcode to be sampled. |
| 5 | Sample one seed from the scanned envelope. |
| 6 | Drop the seed into the corresponding plate well determined by ‘Coordinate’. |
| 7 | ‘Coordinate’ displays the progress of sampling. From time to time, check android device  and confirm that the plate and the application progress are synchronized. |
| 8 | Continue sampling untill all wells are filled except for H11 and H12 (control wells). A special beep sound would indicate that each column is completed. A different beep sound would indicate when the plate is completed. |
| 9 | ‘Coordinate’ automatically stores the plate layout for each sampled plate and can be exported as a .*csv* file. |
| 10 | Cover the plate with cap mat for storage and shipping. |
|  | **See work flow in Supplemental File 1 Figure 1.** |


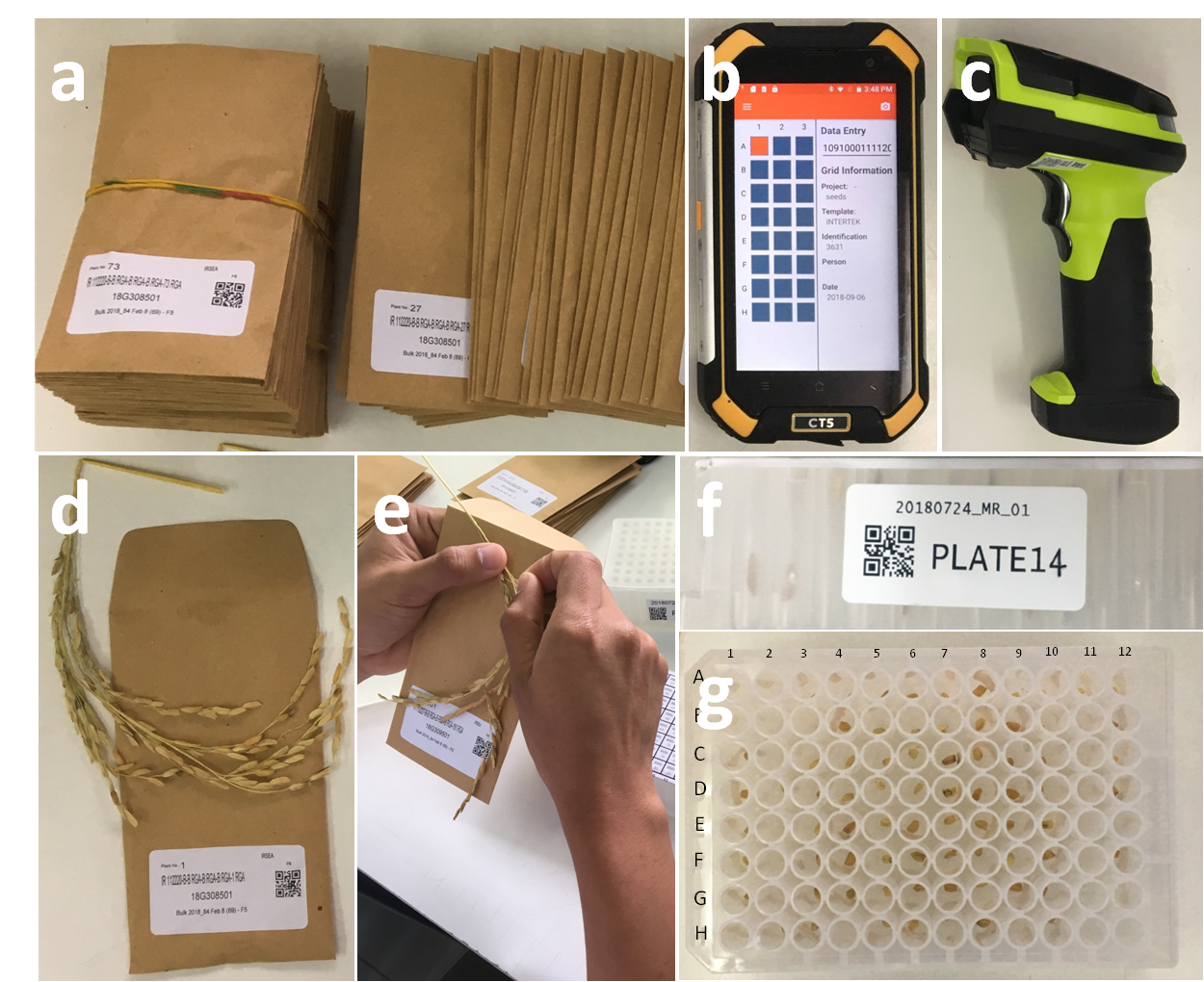


**Additional File 1 Figure S1: single seed based sampling workflow from seed stored in envelopes**. **(a)** Arranged barcoded seed envelopes containing seeds to be sampled - the barcode of each seed envelope is generated and stored in a database that can track the seed source. The single-seed based sampling is coordinated automatically using the application **(b)** ‘Coordinate’ ([www.phenoapps.org](http://www.phenoapps.org)) installed in an Android device (in this case a CT5) and guided using a **(c)** hand-held barcode scanner (in this case Zebra scanner). **(d)** Each seed envelope is scanned **(e)** and a single-seed is collected and placed into the corresponding well defined by ‘Coordinate’ of the **(f)** barcoded plate until the **(g)** whole plate is completed.

| **SINGLE-SEED BASED MARKER ASSISTED SELECTION STRATEGY (*ssb-MASS*):**  **PROTOCOL FOR SAMPLING SEEDS UNDER FIELD/GREENHOUSE CONDITIONS** |
| --- |

| **MATERIALS** | |
| --- | --- |
| 1 | 96-deep-well plates specified by Intertek-Agritech or the genotyping service provider |
| 2 | Hand-held Zebra Scanner (Model: DS3678) or any rugged barcode scanner that is compatible with your barcode labels |
| 3 | CT5 Rugged Phone or any android mobile phone or tablet compatible with ‘Coordinate’ app |
| 4 | Thin grid sticker cover for 96-deep-well plate |
| 5 | 96-deep-well-plate cover mat |

| **PRE-SAMPLING REQUIREMENTS** | |
| --- | --- |
| 1 | Install ‘Coordinate’ ([www.phenoapps.org](http://www.phenoapps.org)) mobile app on the android mobile phone or tablet |
| 2 | Create sampling template using ‘Coordinate’ as specified in the app instructions ([www.phenoapps.org](http://www.phenoapps.org)) skipping wells on the H11 and H12 plate coordinates as determined by Intertek-Agritech (control wells). |
| 3 | Plants or lines to be sampled must be clearly labeled and barcoded. |
| 4 | Seeds to be sampled are preferably between dough to ripening stage of the maturity phase (or between 15 days after panicle initiation to maturity). |
| 5 | Cover plate with sticker paper with printed plate grid. The grid does not necessarily contain any label as it is only used to avoid seeds from coming out of the plate while moving during sampling. |
| 6 | Suspend barcode scanner on ID strap to allow it to hang on neck while sampling. |

| **PROCEDURES** | |
| --- | --- |
| 1 | Connect hand-held Zebra barcode reader and the mobile app via Bluetooth following manufacturer’s specification.  Note: Please see appendices A & B for Zebra Scanner Pairing barcodes. |
| 2 | Open ‘Coordinate’ application on android device. |
| 3 | In the Coordinate application, create a new grid. Coordinate will prompt the user to input the plate name and the username. Alternatively, the user may scan these information from the appropriate barcodes (Plate Barcode & Username Barcode)  Note: See Appendix A for step-by-step instructions |
| 4 | Scan the line or plant barcode to be sampled. |
| 5 | Harvest one ‘healthy’ seed from the tip of one panicle . |
| 6 | Punch a hole through the sticker paper using the seed to place it in the plate well determined by by ‘Coordinate’. |
| 7 | ‘Coordinate’ displays the progress of sampling. From time to time, check the android device  and confirm that the plate and the application progress are synchronized. |
| 8 | Continue sampling untill all wells are filled except for H11 and H12 (control wells). A special beep sound would indicate that each column is completed. A different beep sound would indicate when the plate is completed. |
| 9 | ‘Coordinate’ automatically stores the plate layout for each sampled plate and can be exported as a .*csv* file. |
| 10 | Peel sticker paper carefully to ensure no paper gets into the wells and replace with cap mat for storage and shipping. |
|  | **See work flow in Supplemental File 1 Figure 2 and Figure 3.** |


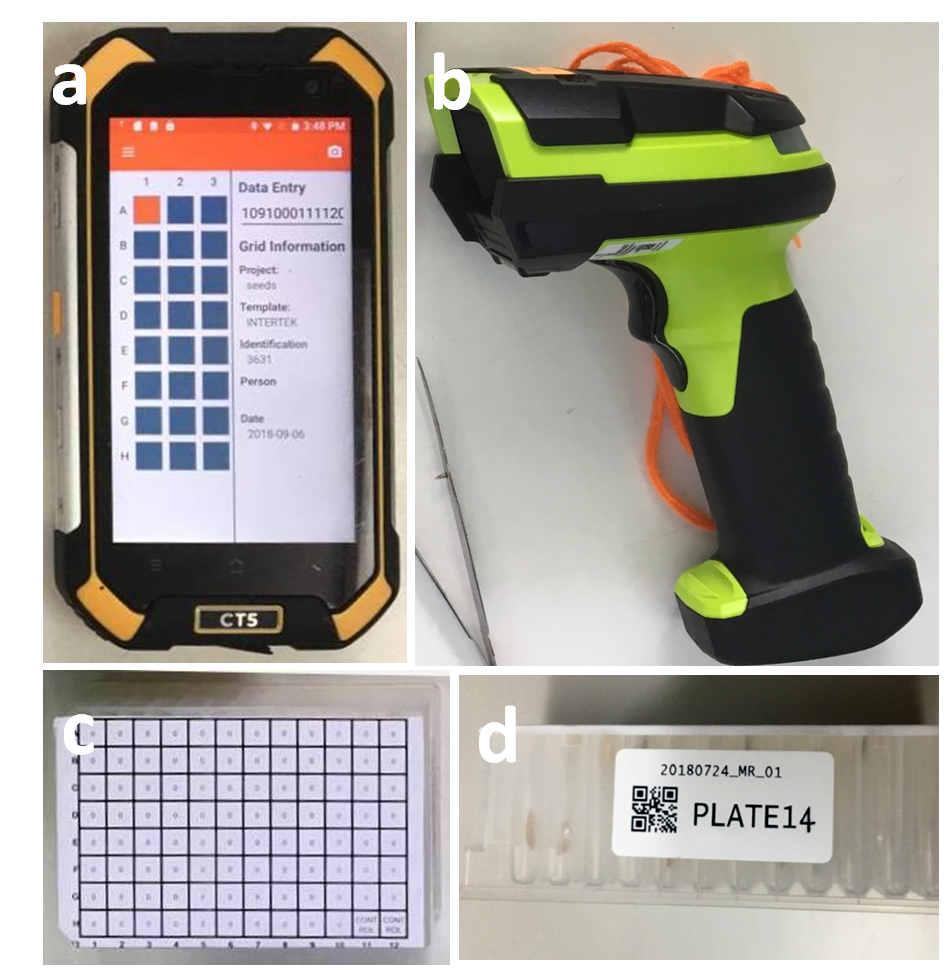


**Additional File 1 Figure S2: single-seed based sampling set up for plants grown under field or greenhouse conditions.** Field sampling materials to be prepared include **(a)** Android device with ‘Coordinate’ application installed, **(b)** hand-held wireless barcode scanner **(c)** 96-deep-well plate - specified by Intertek-AgriTech or other genotyping service provider and covered with sticker paper printed with well grid. **(d)** The plate should be barcoded for ease of tracking.


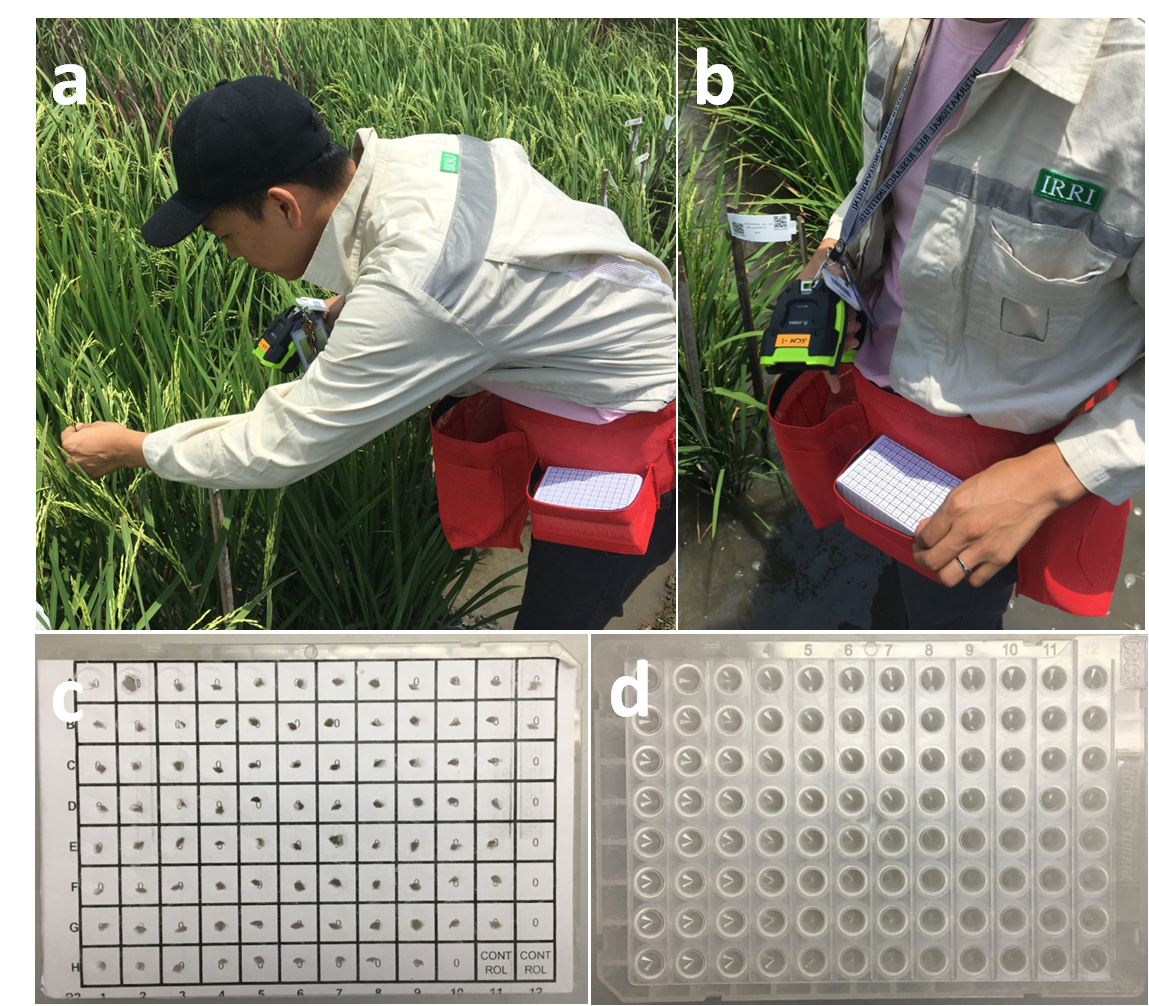


**Additional File 1 Figure S3: In-field or greenhouse single-seed based sampling protocol.** **(a)** Line or plant barcode is scanned followed by sampling a ‘healthy’ single-seed from the panicle. **(b)** Insert seed into the plate well determined by ‘Coordinate’ application. Repeat the whole process until **(c)** the whole plate is completed. **(d)** After sampling, the sticker paper is carefully removed and replaced with cap mat for storage or shipping.

Appendix S1. **STEP BY STEP GUIDE TO USING THE ZEBRA SCANNER AND CT5 (or Other Android Phone with Bluetooth Functionality)**

**Pairing and Using Ct5 and Zebra Scanners for Plate Sampling**

1. Put batteries in Zebra scanner. It should emit a sound.
2. Scan “Set Default Barcode” or “Set Factory Default”
3. Scan “BT HID Slave” (opens Zebra Scanners Bluetooth, enabling CT5 to pair with it)
4. Turn on CT5 and open Bluetooth feature. Wait for the serial number of the Zebra Scanner to show, then pair CT5 with the respective Zebra scanner. You will hear a beep sound once connected.
5. Scan “Add Enter Key” barcode (enables auto enter key)
6. Scan “Battery Status Low” barcode (enables beep notification when scanner battery is low = 4 beeps)
7. In CT5, open “Coordinate” app
8. In “Coordinate” app, create a grid, then select “Create a project for this grid”.
9. Scan “Project Name”, press OK
10. Select Load Template then select “INTERTEK”
11. Scan Plate ID in the “Identification tab; Scan “Name”; Tap Create
12. You can now start scanning. For every 8 samples, a beep notification will be heard. After all 94 samples were collected, a success tone beep will be heard. Click Ok.
13. Create new grid, select “Add to [name of project]”. Continue number 11-13.
14. Once all plates have been collected, export project by tap “export” under Project Menu, select Project then click OK.
15. Double check the exported project by going to this path:

File Manager/Internal Storage/Coordinate/Export/INTERTEK/[Project Name]

1. Once finished, scan “Unpairing” barcode. Turn off Bluetooth in CT5

Other Useful Barcodes

- Volume (High, Medium, Low) = scan to adjust the beep sound of your scanner
- No Sample = scan if you need to skip a certain well

Appendix S2. Printable Barcodes for Zebra Scanner Pairing


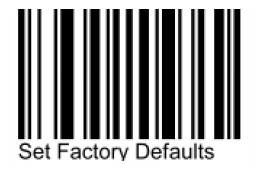


1)


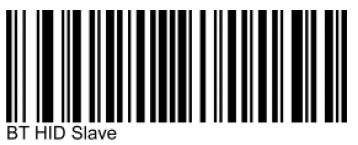


2)


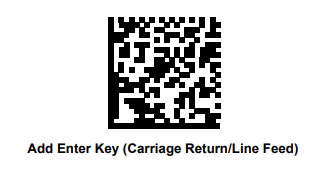


3)


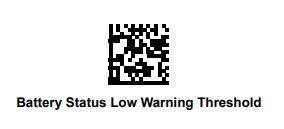


4)


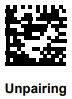


5)


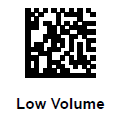

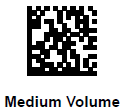

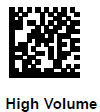


6)


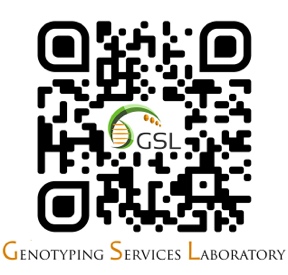


7)

1. Resets the state of the scanner
2. Opens the bluetooth functionality of the scanner and allows it to pair to any android device
3. Automatically adds an Enter key after scanning a barcode
4. Allows scanner to alert user once the battery of the scanner is low
5. Unpairs the scanner to it’s paired android device
6. Allows the user to define the volume of the scanner
7. Leads the user to the GSL website
